# Assessment of genetic diversity and genetic relationships of farm and laboratory quail populations in Japan using microsatellite DNA markers

**DOI:** 10.1002/vms3.328

**Published:** 2020-07-24

**Authors:** Mitsuo Nunome, Rie Yoshioka, Takuro Shinkai, Katsutoshi Kino, Yoichi Matsuda

**Affiliations:** ^1^ Avian Bioscience Research Center Graduate School of Bioagricultural Sciences Nagoya University Nagoya Japan; ^2^ Aichi Agricultural Research Center Nagakute Japan; ^3^ Laboratory of Avian Bioscience Department of Animal Sciences Graduate School of Bioagricultural Sciences Nagoya University Nagoya Japan

**Keywords:** breeding, genetic assessment, Japanese quail, molecular phylogenetic tree, SSR, STRUCTURE plot

## Abstract

**Background:**

The Japanese quail (*Coturnix japonica*) is an important poultry species owing to their high economic efficiency and biological advantages. The genetic diversity of farm quail populations has rarely been studied.

**Objectives:**

This study aimed to assess the genetic diversity of farm quail populations and their genetic relationships, which could provide important information for designing breeding programmes to maintain egg and/or meat production efficiency.

**Methods:**

Molecular phylogenetic and STRUCTURE analyses were conducted for seven farm populations and six laboratory lines using 50 microsatellite markers previously developed by us.

**Results:**

The genetic diversity within each farm population was relatively high despite long‐term breeding within closed colonies. However, the genetic variation between populations was absent. Twenty highly polymorphic markers, selected based on *Ne*, *He* and *F_ST_* values, enabled the construction of reliable phylogenetic trees and STRUCTURE plots.

**Conclusions:**

In the farm populations analysed in the present study, gene flow between genetically distant populations is needed to restore genetic diversity between farm populations, which could exploit heterosis and decrease the risk of inbreeding depression. Our findings demonstrate that these markers are useful for examining the genetic structure of farm quail populations.

## Graphical abstract

We conducted genetic monitoring for six farm populations in Aichi prefecture, Japan, and five laboratory lines, whose breeding histories are well recorded, using 48 microsatellite markers. We estimated the genetic diversity of the farm populations and their genetic relationships by molecular phylogenetic and STRUCTURE analyses, which provided important information to obtain suitable genetically distinct candidates for cross‐mating to restore genetic diversity among populations.

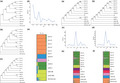

## 1 INTRODUCTION

The Japanese quail (*Coturnix japonica*) is the smallest poultry species used for meat and egg production. Because of their high economic efficiency, rapid sexual maturation and resistance to environmental stresses and disease, Japanese quail have attracted increasing attention as poultry species, second to the chicken, especially in developing countries (Bakoji et al., 2013; Nasar, Rahman, Hoque, Talukder, & Das, 2016; Santhi & Kalaikannan, 2017). Several studies have examined the social and economic status of farm quail (Das et al., 2008; Redoy, Shuvo, & Al‐Mamun, 2017; Saka, Oyegbami, Okere, Omole, & Fayenuwo, 2018), their blood biochemical profiles and biological properties (Mnisi et al., 2018; Al‐Tikriti, 2019) and the genetic diversity in farm populations (Bai, Pang, Zhang, Yun, & Qi, 2016; Rifki, Dewanti, Widyas, & Cahyadi, 2018; Shimma & Tadano, 2019). This information can be used to improve the production efficiency of farm quail populations.

In Japan, their commercial use started at the beginning of the 20th century (Wakasugi, 1984), and commercial quail were first exported to the United States and Europe in the 1930s (Minvielle, 2004). Following a severe decline in Japan during World War II, domestic quail populations were re‐established from a few surviving individuals (Wakasugi, 1984; Yamashina, 1961). The re‐established commercial quail were again exported and rapidly distributed worldwide. Most domestic Japanese quail that are currently bred worldwide are thought to have been derived from the populations that were re‐established in Toyohashi city, Aichi prefecture, Japan, after World War II (Wakasugi, 1984; Yamashina, 1961). Toyohashi is still a centre of quail egg production, accounting for approximately 60% of commercial egg production in Japan. Even though 4.62 million birds were raised on 83 farms in Toyohashi area in 1980, these numbers decreased to 1.76 million birds raised on 12 farms by 2018 (unpublished data from the Department of Agriculture, Forestry, and Fisheries, Aichi Prefectural Government). However, the average number of quail per farm has increased from around 56,000 in 1974 to around 147,000 in 2018. Furthermore, farmers in Aichi prefecture have generally preferred closed breeding within a colony, without introducing sire quail from other farms for many years. Japanese quail are known to be very susceptible to inbreeding depression (Shinjo, Mizuma, & Nishida, 1971; Sittmann, Abplanalp, & Fraser, 1966), raising concerns for farmers that productivity will decrease as a consequence of the long‐term breeding within a closed colony. One way to escape inbreeding depression is cross‐mating with genetically distant quail, which is an effective method of restoring genetic diversity in farm quail populations. For this, monitoring the genetic diversity and/or autozygosity within populations, as well as the genetic variation between populations, are required for determining which populations should be introduced for cross‐mating.

In our previous study (Nunome et al., 2017), genetic characterisation was carried out for 19 domestic populations of Japanese quail, consisting of nine laboratory lines, six meat‐type quail lines, one commercial population and three wild quail populations from East Asia, using 50 highly variable microsatellite DNA markers. A phylogenetic tree constructed with the markers clearly represented the breeding history of laboratory lines established in the last 50 years, indicating that the microsatellite markers are effective for estimating the genetic diversity of farm quail populations and their genetic relationships. Recently, Shimma and Tadano (2019) examined genetic diversity of 12 laying‐type quail lines collected from nine farms in five prefectures in Japan, using 50 microsatellite markers. In the study, although four farms in Aichi prefecture showed genetic diversity as high as farm quail in the other prefectures, they could not find clear genetic differences among lines.

In this study, to assess the genetic diversity within and between farm populations of Japanese quail, we conducted genetic monitoring for six farm populations from Aichi prefecture, and five laboratory lines using 50 microsatellite markers that were developed previously (Tadano et al., 2014). Then, we performed molecular phylogenetic and STRUCTURE analyses for seven farm populations and six laboratory lines, including our previous data of one farm population from Saitama prefecture and one laboratory line (the wild‐derived line) to find genetically distinct candidates that can be used for restoring the genetic diversity of the farm quail populations. Then, we assessed the effective number of highly polymorphic markers that are needed to accurately estimate the genetic structure of farm quail populations.

## 2 MATERIALS AND METHODS

### 2.1 Specimens and genomic DNA extraction

Whole blood samples were collected from wing veins of 499 individuals from the following 11 populations (Table S1): four laboratory quail lines (AARC‐B, ‐C, ‐BB and ‐WW) from Aichi Agricultural Research Center (AARC) (AARC‐B and AARC‐C have been maintained for 25 generations and AARC‐BB and AARC‐WW for 15 generations); one laboratory line, DY, that derived from a dominant yellow mutant which was found in a farm of Toyohashi in 1963 (this mutant may have been mated with commercial quail that derived from an unknown farm and then has been maintained as a closed colony for nearly half a century); four farm populations from Toyohashi city (Farms A–D) and one population from each of two neighbouring cities (Toyokawa, Farm E; Tahara, Farm F); Genomic DNA was extracted from 20 µl of whole blood using 300 µl of DNAzol BD reagent (Molecular Research Center, Tokyo, Japan). For construction of phylogenetic trees and STRUCTURE analysis, the genotyping data of the following two quail populations that were reported in our previous study were included (Tadano et al., 2014): 57 commercial quail from a farm in Saitama prefecture and 40 quail from a wild‐derived line that was established from wild‐captured quail at the National Institute of Genetics, Japan, and is now maintained at the National Institute of Livestock and Grassland Science (NILGS), National Agriculture and Bio‐oriented Research Organization, Japan.

### 2.2 PCR amplification

PCR amplification of the 50 microsatellite markers used in our previous study (Nunome et al., 2017) was performed using a 10‐µl reaction mix containing approximately 50 ng of genomic DNA, 10 pmol of each primer and 5 µl of Taq Gold 360 Master Mix (Thermo Fisher Scientific‐Applied Biosystems). The following PCR cycling conditions were used: initial denaturation at 95°C for 10 min, followed by 42 cycles at 95°C for 30 s, 50°C or 55°C for 30 s, and 72°C for 25 s, and a final extension at 72°C for 5 min. The nucleotide sequences and suitable annealing temperatures of all the primer sets were provided in our previous study (Tadano et al., 2014). Amplicons were electrophoresed with Hi‐Di formamide (Thermo Fisher Scientific) and the GeneScan 600 LIZ Size Standard (Thermo Fisher Scientific) using the ABI PRISM 3130 Genetic Analyzer (Thermo Fisher Scientific). Allele size was determined using GENEMAPPER version 4.1 (Thermo Fisher Scientific).

### 2.3 Estimation of genetic diversity within populations

The total number of alleles (*NA*) per marker was counted using MICROSATELLITE ANALYSER 4.05 (Dieringer & Schlötterer, 2003). The mean number of effective alleles (*Ne*, the number of equally frequent alleles at a marker), observed heterozygosity (*Ho*), expected heterozygosity (*He*), fixation indices (*F_IS_*, *F_ST_* and *F_IT_*) and the chi‐square statistic for Hardy‐Weinberg equilibrium (*HWE*) were calculated for each marker using GENALEX 6.5 (Peakall & Smouse, 2012). Polymorphic information content (PIC) was calculated using CERVUS 3.0.7 (Kalinowski, Taper, & Marshall, 2007). Null allele frequency (*NAF*) was estimated based on the Expectation Maximisation (EM) algorithm (Dempster, Laird, & Rubin, 1977) using the FreeNA software (Chapuis & Estoup, 2006). Allelic richness (*AR*) in each population was calculated using MICROSATELLITE ANALYSER 4.05 (Dieringer & Schlötterer, 2003). The genetic diversity of each population was estimated based on the mean genetic distance (*MGD*) between individuals within a population determined using the relatedness analysis conducted with GENEALEX 6.5 (Peakall & Smouse, 2012). The codominant genetic distance (Smouse & Peakall, 1999) was used to calculate the *MGD* and the upper and lower limits of the 95% confidence interval of the *MGD* were determined using 1,000 bootstrap replicates.

### 2.4 Estimation of genetic relationships among populations

Pairwise genetic distances between populations were calculated using MICROSATELLITE ANALYSER 4.05 (Dieringer & Schlötterer, 2003) based on three different genetic distances, namely, Nei's angular genetic distance based on allele frequencies (Da; Nei, Tajima, & Tateno, 1983), the genetic distance based on the proportion of shared alleles (Dps; Bowcock et al., 1994) and the Cavalli‐Sforza chord distance based on allele frequencies (Dc; Cavalli‐Sforza & Edwards, 1967). The wild‐derived line was used as an outgroup to reconstruct the phylogenetic trees. The bootstrap values were calculated with 1,000 replicates. Bayesian clustering analysis was performed to examine genetic relationships among 13 populations using STRUCTURE 2.3 (Pritchard, Stephens, & Donnelly, 2000). Log probability values (Ln P[D]) were estimated for each K from 1 to 14 with sampling periods of 100,000 MCMC generations after burn‐in periods of 300,000 generations, using the admixture and the correlated allele frequency models (Porras‐Hurtado et al., 2013). Twenty independent MCMC runs were performed for each K. Among 20 runs, those that showed obviously large values for the variance of Ln likelihood were discarded because MCMC simulations do not sufficiently converge on an optimal Ln likelihood value when the variance is large. The results were averaged using CLUMPP 1.1.2 (Jakobsson & Rosenberg, 2007) and graphed with DISTRUCT 1.1 (Rosenberg, 2004). The optimal K value was estimated using the Evanno method (Evanno, Regnaut, & Goudet, 2005), implemented in STRUCTURE HARVESTER 0.6.94 (Earl & vonHoldt, 2012).

### 2.5 Selection of 20 informative microsatellite markers

First, phylogenetic trees were constructed for 13 populations with three genetic distances (Da, Dps, and Dc) using 48 markers, excluding two monoallelic markers (see the Results section), and we determined the genetic distance needed to construct the phylogenetic tree that reflected the breeding histories of the populations most accurately. Then, sets of the top 20 markers were selected for each *Ne*, *He* and *F_ST_* that satisfied the conditions of not significantly deviating from Hardy‐Weinberg equilibrium (*HWE*) and presenting null allele frequencies less than 0.1 (the markers shown in bold in Table S1). The molecular phylogenetic trees were constructed with each set of 20 markers using Cavalli‐Sforza chord distance based on allele frequencies (Dc) (see the details in the Results section). STRUCTURE analysis was also performed using three sets of 20 markers, and the results were compared with those generated using 48 markers.

## 3 RESULTS

### 3.1 Genetic diversity of quail populations

Two markers, NGJ0023 and NGJ0045, were monoallelic (Table S1), and the remaining 48 were used for subsequent population genetic analyses. The highest number of alleles was observed for NGJ0050 (*NA* = 23) and the lowest for NGJ0007, NGJ0022 and NGJ0044 (*NA* = 3) (Table S1). The *Ne* was the highest for NGJ0050 (4.76) and the lowest for NGJ0042 (1.33). The PIC ranged from 0.27 (NGJ0044) to 0.87 (NGJ0018). The *He* and *F_ST_* values ranged from 0.27 (NGJ0039) to 0.77 (NGJ0050) and from 0.05 (NGJ0037, NGJ0044) to 0.63 (NGJ0039), respectively. Significant deviation from the *HWE* was found for 10 markers (NGJ0003, NGJ0007, NGJ0014, NGJ0015, NGJ0019, NGJ0033, NGJ0039, NGJ0042, NGJ0046 and NGJ0050). Null allele frequencies ranged from 0 (NGJ0044) to 0.35 (NGJ0039).

Genetic diversity indices for each population are shown in Table 1. The mean number of alleles per locus (*MNA*) ranged from 2.82 for the DY line to 5.52 for the Farm A line. The *AR* ranged from 2.78 (DY) to 4.90 (Farm D). The lowest *Ne* and *Ho* values were observed for the DY line (*Ne* = 1.95, *Ho* = 0.37), whereas the highest values were observed for Farm D (*Ne* = 2.99, *Ho* = 0.55). Six farm populations from Aichi prefecture (Farms A‒F) showed *F_IS_* values of 0.10 on average, ranging from 0.08 (Farm D) to 0.13 (Farm F). The Saitama population had a marginally higher *F_IS_* value (0.12). The *MGD* values for Farms A‒*F* (66.51 on average) were similar to those obtained for the Saitama population (67.01), but were relatively lower (51.87 on average) in four AARC laboratory lines (AARC‐B, ‐C, ‐BB, and ‐WW), as were the *F_IS_* values (0.02 on average). These results suggest that genetic diversity was not especially low in all the farm populations from Aichi prefecture (Farms A‒F) compared with the farm population from Saitama prefecture. However, genetic variability in the DY and four AARC laboratory lines was substantially lower due to their small population sizes; the DY line has maintained as a closed population which are generally composed of about 30 males and 50 females per generation, and each of four AARC lines has consisted of around 150 males and 350 females per generation.

**TABLE 1:** Genetic diversity of 13 quail populations estimated using 48 microsatellite markers

Population or line	*n*	*MNA*	*AR*	*Ne*	*Ho*	*He*	*F_IS_*	*MGD* (95% confidence interval)
Farm A	48	5.52	4.75	2.92	0.53	0.59	0.097	66.48 (66.96‒65.99)
Farm B	48	5.36	4.79	2.96	0.54	0.60	0.109	69.18 (69.61‒68.76)
Farm C	48	5.30	4.49	2.77	0.51	0.56	0.100	63.96 (64.42‒63.50)
Farm D	48	5.46	4.90	2.99	0.55	0.59	0.082	65.52 (65.99‒65.04)
Farm E	48	5.26	4.63	2.85	0.54	0.58	0.089	64.79 (65.23‒64.37)
Farm F	14	4.34	4.65	2.64	0.44	0.52	0.131	69.13 (70.95‒67.21)
Saitama	57	5.32	4.77	2.86	0.50	0.56	0.120	67.01 (67.47‒66.58)
DY	45	2.82	2.78	1.95	0.37	0.41	0.088	47.53 (48.08‒46.91)
AARC‐B	50	3.30	3.31	2.24	0.48	0.49	0.025	52.05 (52.53‒51.56)
AARC‐C	50	3.44	3.43	2.17	0.45	0.46	0.018	50.81 (51.33‒50.24)
AARC‐BB	50	3.62	3.59	2.25	0.46	0.47	0.016	52.10 (52.56‒51.63)
AARC‐WW	50	3.42	3.44	2.24	0.49	0.50	0.015	52.50 (52.98‒51.97)
Wild‐derived	40	4.08	3.86	2.60	0.52	0.56	0.085	61.64 (62.18‒61.08)

Abbreviations: AARC‐B, ‐C, ‐BB and –WW, laboratory lines from Aichi Agricultural Research Center; *AR*, allelic richness; DY, laboratory line derived from a dominant yellow mutant that was found on a farm in Toyohashi in 1963; Farm E, farm population from Toyokawa; Farm F, farm population from Tahara; Farms A–D, farm populations from Toyohashi; *F_IS_*, Fixation Index = (*He* − *Ho*)/*He*; *He*, expected heterozygosity; *Ho*, observed heterozygosity; *MGD*, mean genetic distance between individuals within a population; *MNA*, mean number of alleles per locus; *n*, number of individuals; *Ne*: mean number of effective alleles per locus = 1/(Sum pi^2), where Sum pi^2 is the sum of the squared population allele frequencies; Saitama, commercial quail from a farm in Saitama prefecture; Wild‐derived, laboratory line established from wild‐captured quail.

### 3.2 Genetic relationships between quail populations

**FIGURE 1:**
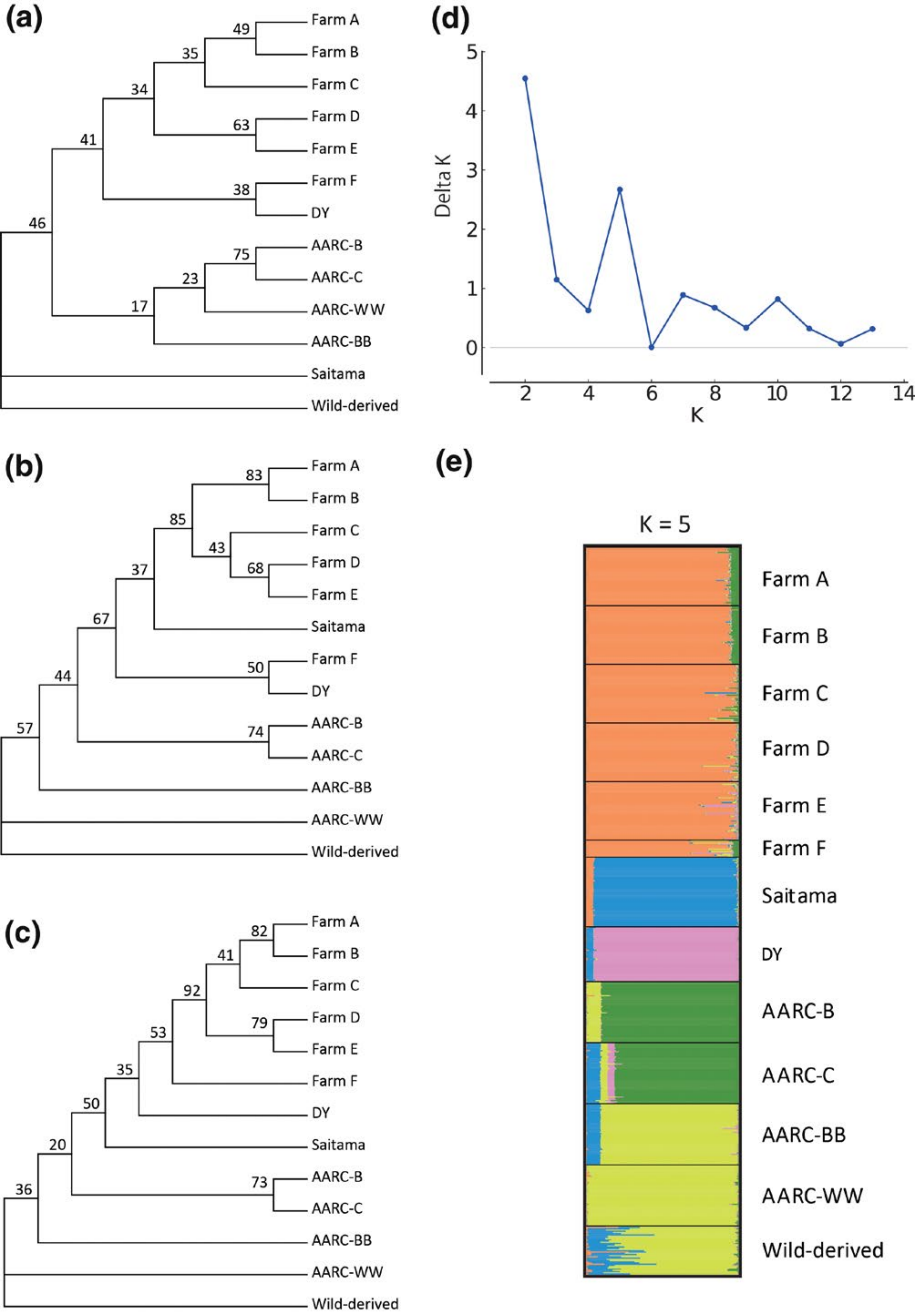
Neighbour‐joining trees and STRUCTURE plot of 13 quail populations based on 48 microsatellite markers. (a–c) Neighbour‐joining trees constructed with Nei's genetic distance based on allele frequencies (Da; Nei et al., 1983) (a), genetic distance based on the proportion of shared alleles (Dps; Bowcock et al., 1994) (b) and Cavalli‐Sforza chord distance based on allele frequencies (Dc; Cavalli‐Sforza & Edwards, 1967) (c). (d and e) Delta *K* values at *K* = 2 to *K* = 13 generated by STRUCTURE HARVESTER (d) and STRUCTURE plot at *K* = 5 (e). Each horizontal bar represents an individual, and each colour indicates the probability of belonging to one of the genetic clusters

Three phylogenetic trees for 13 populations based on Da (Figure 1a), Dps (Figure 1b), and Dc (Figure 1c) genetic distances were constructed. The topologies were similar for the three trees. Five farm populations (Farms A‒E) from Toyohashi and Toyokawa were monophyletic in all trees, and monophyly was also observed for all the farm populations from Aichi prefecture (Farms A‒F) in the Dc tree. Furthermore, the DY line showed a close genetic relationship with Farms A‒F in all three trees. In contrast, four AARC laboratory lines (AARC‐B, ‐C, ‐BB and ‐WW) were genetically clearly distinct from the farm populations from the Aichi prefecture in the three trees. Three laboratory lines (AARC‐BB, AARC‐WW and wild‐derived) were phylogenetically distant from the farm populations in the Dps and Dc trees. The Saitama population was positioned as an outgroup with the wild‐derived line in the Da tree, whereas it was nested within the farm populations from Aichi prefecture (Farms A‒F) in the Dps tree. Only the Dc tree placed the Saitama population as a sister lineage of the clade consisting of the DY line and the farm populations from Aichi prefecture.

STRUCTURE HARVESTER analysis indicated that the highest Delta K value was 2 (Figure 1d); however, the STRUCTURE plot at *K* = 2 only separated farm and farm‐derived populations (Farms A‒F, DY and Saitama) and the rest (four AARC lines and the wild‐derived line) (data not shown), which was not informative for our research. Therefore, we constructed the STRUCTURE plot with the second highest *K* value (*K* = 5) because it was more informative. All six farm populations from Aichi prefecture (Farms A‒F) clustered together (shown in orange) (Figure 1e) and were clearly separated from the farm quail population from Saitama. The AARC‐B and AARC‐C laboratory lines clustered in one group (green), whereas the AARC‐BB, AARC‐WW and wild‐derived lines were assigned to the same cluster (yellow). The DY line and Saitama population clustered individually.

### 3.3 Genetic relationships of quail populations estimated using 20 selected markers

**FIGURE 2:**
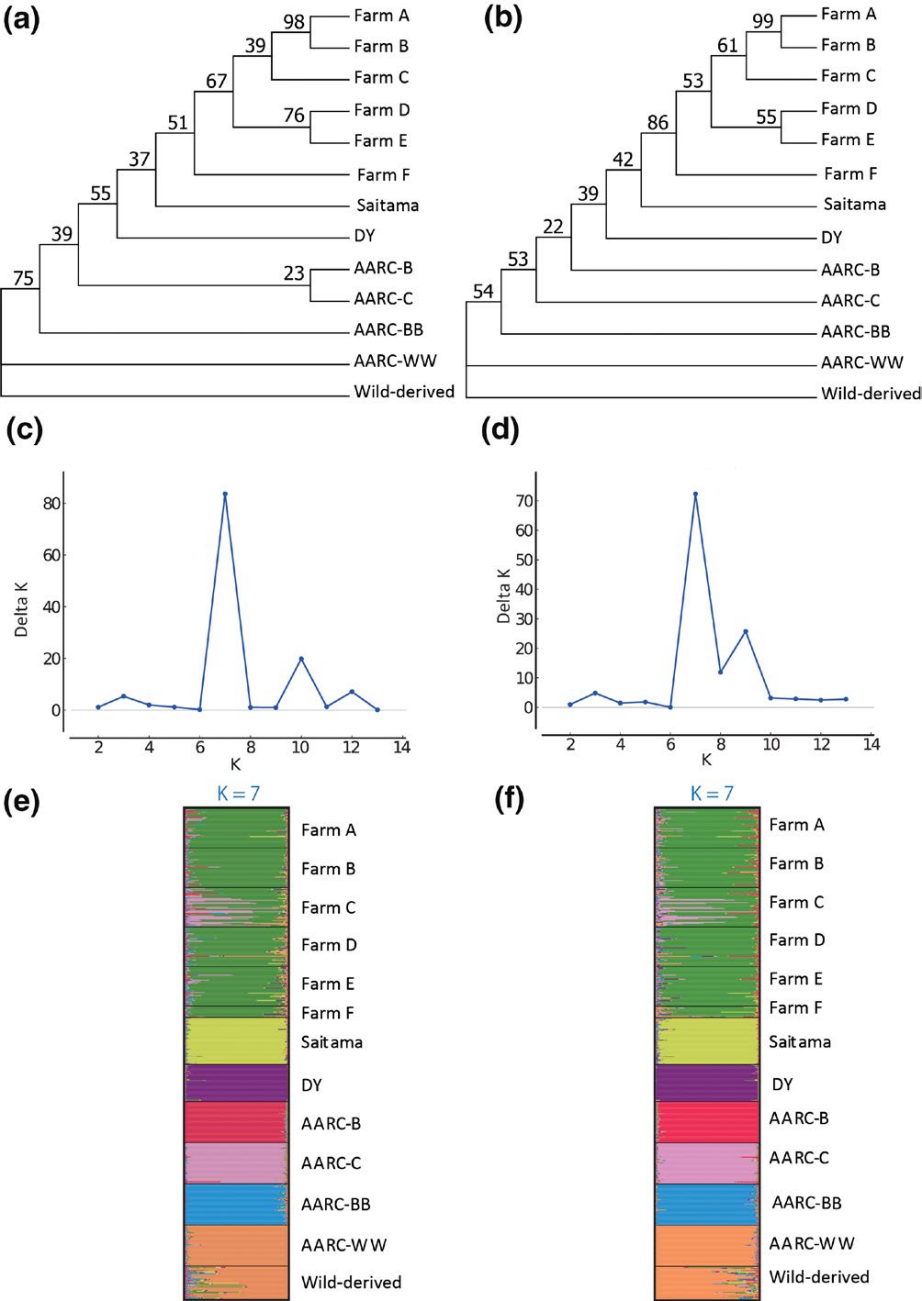
Neighbour‐joining trees and STRUCTURE plots of 13 quail populations based on 20 highly polymorphic microsatellite markers. (a and b) Neighbour‐joining trees based on the Cavalli‐Sforza chord distance, constructed with two sets of 20 microsatellite DNA markers that showed high *Ne* and *He* values (NeHe‐20) (a), and high *F_ST_* values (F_ST_‐20) (b). (c–f) Delta *K* values at *K* = 2 to *K* = 13 and STRUCTURE plots at K = 7 with NeHe‐20 (c, e) and F_ST_‐20 (d and f) maker sets. Each horizontal bar represents an individual, and each colour indicates the probability of belonging to one of the genetic clusters

Twenty highly polymorphic markers that were selected based on the *Ne* and *He* values were the same (Table S1); therefore, we conducted phylogenetic and STRUCTURE analyses using two sets of 20 markers selected based on the *Ne* and *He* values (NeHe‐20) and the *F_ST_* value (F_ST_‐20), both of which covered 15 chromosomes. We note that F_ST_‐20 included four markers with *F_ST_* values less than 0.10 (NGJ0011, NGJ0013, NGJ0022 and NGJ0027). Because the Dc genetic distance produced the most reliable tree with the 48 markers (see the details in the Discussion), the phylogenetic analyses with NeHe‐20 and F_ST_‐20 were performed using the Dc genetic distance. The NeHe‐20 and F_ST_‐20 phylogenetic trees were largely similar (Figure 2a and b), and were both also similar to the tree constructed with the 48 markers (Figure 1c). Both trees also showed close genetic relationships among the six farm populations from Aichi prefecture, as well as the monophyly of three Toyohashi farms (Farms A − C). Four AARC lines (AARC‐B, ‐C, ‐BB and ‐WW) were clearly separated from six Aichi farm populations (Farms A–F), as well as from the DY and Saitama populations. A close genetic relationship between the AARC‐WW and wild‐derived lines was also shown in both trees.

The STRUCTURE analysis showed that the highest Delta K value for both sets of 20 markers was at *K* = 7 (Figure 2c and d), and the genetic structure patterns of 13 populations were very similar between the two marker sets (Figure 2e and f). Six Aichi farm populations clustered together, even though several quail from Farm C showed genetic affinities with the AARC‐C line. However, the DY, AARC‐B, AARC‐C, AARC‐BB lines and the Saitama population, each formed a single cluster. The remaining cluster consisted of the AARC‐WW and wild‐derived lines.

## 4 DISCUSSION

### 4.1 Genetic diversity of farm quail populations

This study revealed that the genetic diversity of farm quail populations in Toyohashi (Farms A–D), Toyokawa (Farm E) and Tahara (Farm F) in Aichi prefecture was as high as that observed in the farm population from Saitama prefecture. Furthermore, their *AR* and *Ho* were also similar to those of several other farm populations in Japan (Shimma & Tadano, 2019). Forty‐five of 50 microsatellite markers used in Shimma and Tadano (2019) were the same as those used in this study. These results indicate that the genetic diversity of quail farm populations in Aichi prefecture has not decreased despite long‐term breeding within closed colonies. In each of the four Toyohashi farms, 10,000 to 150,000 quail are generally maintained as egg‐laying populations and each population is produced by 2,000 to 9,000 dams. The dams and the egg‐laying populations are renewed every 6 months and every year, respectively. Their population sizes seem to be large enough to prevent the loss of genetic diversity; however, the genetic relationships among the six farm populations from Aichi prefecture were very close (Farms A–F). The quail farms in Aichi prefecture have occasionally (once every 5 or more years) introduced sires from other farms to recover the genetic diversity of the populations; however, the change in sires is generally only carried out from a limited number of farms within the same region. For example, Farms A, B, and C introduced sires from the same farm (Farm G; this population was not included in this study) in 2013, 2012 and 2011, respectively. Results for the phylogenetic trees and STRUCTURE plots suggest that the genetic divergence between the farm populations will not be recovered by the introduction of sires from other farm populations of Toyohashi. Therefore, to restore the genetic divergence, sires must be introduced from more genetically distant populations. To recover the genetic diversity in quail farms, the Aichi Prefectural Government has recently introduced two quail lines, AARC‐BB and AARC‐WW, into Toyohashi farms, which are used for crossbreeding with the farm quail. The AARC‐BB and AARC‐WW lines are genetically more distant from the farm populations, compared with the other two lines (AARC‐B and ‐C). In addition, the two lines produce many eggs that have similar characteristics (colour, weight, etc.) to those of the farm quail. AARC‐BB is a mutant line, with Z‐linked recessive incomplete albinism; therefore, the sex of descendants can easily be distinguished by plumage colours (i.e. when sires of AARC‐BB are crossed with dams of farm quail with the wild‐type plumage).

### 4.2 Genetic relationships and breeding histories of farm and laboratory quail populations

The phylogenetic trees constructed with the 48 microsatellite markers showed very close genetic relationships among six populations from Aichi prefecture (Farms A–F). Moreover, the close relationship of the DY line with the six farm populations shown in the Da and Dc phylogenetic trees was compatible with the breeding history of the DY line that originated from a farm population in Toyohashi in 1963. The AARC‐WW line is a direct descendant from the wild‐derived line introduced from the NILGS. The AARC‐B line was established by crossing the wild‐derived line with a laboratory line introduced from Shinshu University, Japan, whereas the AARC‐C line was established by crossing the wild‐derived line with commercial quail. Consequently, these three lines share a common ancestor (the wild‐derived line), which explains their close genetic relationships. The AARC‐BB line descended from the NIES‐Br line originating from a meat‐type quail population with brown plumage that was imported from Brazil and maintained at the National Institute of Environmental Studies (NIES), Japan. STRUCTURE analysis of 15 laboratory lines in our previous study (Nunome et al., 2017), including the NIES‐Br and the wild‐derived lines, showed a high genetic affinity between the NIES‐Br and wild‐derived lines; this may explain why the AARC‐BB line is also closely related to the other three AARC laboratory lines (AARC‐B, AARC‐C and AARC‐WW) that share a common origin from the wild‐derived line.

The phylogenetic trees and STRUCTURE plots constructed with the 48 markers clearly supported the breeding histories of the farm and laboratory quail populations examined in this study, even though null allele frequencies were higher than 0.20 for five of the markers. In particular, the tree based on the Dc genetic distance matched better to known histories than those based on the Da and Dps genetic distances. The positions of the DY line and Saitama population and the monophyly of the six farm populations from Aichi prefecture on the Dc tree indicated that the Dc tree is more reliable. In our previous study on the genetic characterisation for laboratory chicken lines, the Dps genetic distance constructed less realistic phylogenetic trees than that of Da genetic distance (Nunome et al., 2019). The Dc and Da genetic distances are known to be better estimators for topology construction (Takezaki & Nei, 1996). Recently, a simulation study by Sere, Thevenon, Belem, and De Meeûs (2017) confirmed that the Dc genetic distance is the most robust, even in the presence of null alleles, which is similar to that observed by Carlsson (2008), who revealed that STRUCTURE analysis also produces reliable results in the presence of null alleles. As to the STRUCTURE analysis, 300,000 MCMC generations were discarded as burn‐in periods in every run, which was three times larger than the length of burn‐in periods in Shimma and Tadano (2019). Besides, all results of MCMC simulations which showed large variance values of Ln likelihood were removed from the subsequent steps of the STRUCTURE analysis. These data processing steps might contribute to the reconstruction of breeding history of farm quail populations and the detection of genetic differences between farm quail populations in Aichi and Saitama prefectures. Thus, the phylogenetic tree based on the Dc genetic distance and STRUCTURE plots demonstrates the breeding histories of quail populations with reduced influence of null alleles.

### 4.3 Validity of 20 microsatellite markers for genetic assessment

Microsatellites are one of the most popular markers for estimating the genetic diversity in animal and plant populations. Microsatellite markers for Japanese quail were developed comprehensively in previous studies (Kayang et al., 2000, 2002, 2004, 2006; Mannen et al., 2005). They have been used to discover gene loci associated with plumage colour, body weight, meat quality and egg production (Minvielle et al., 2005, 2006; Miwa et al., 2005; Ori, Esmailizadeh, Charati, Mohammadabadi, & Sohrabi, 2014; Rezvannejad, Yaghoobi, & Rashki, 2014; Tavaniello et al., 2014; Moradian et al., 2016; Knaga et al., 2018); however, the chromosomal position of these markers had remained undetermined. We recently developed more than 100 microsatellite DNA markers from Japanese quail based on the whole‐genome sequence, covering chromosomes 1 to 28 and the Z and W sex chromosomes (Kawahara‐Miki et al., 2013). In this study, we further selected two sets of 20 microsatellite markers (NeHe‐20 and F_ST_‐20) that produced reliable phylogenetic trees and STRUCTURE plots. The STRUCTURE plots constructed with NeHe‐20 and F_ST_‐20 may be more informative than that produced with 48 microsatellite markers. First, the Farm C population showed partial genetic affinity with the AARC‐C line, supporting that sires from the AARC‐C line were introduced into Farm C in 2012; and second, the AARC‐WW and wild‐derived lines, which have the same origin, were grouped into a single genetic cluster that did not include the AARC‐BB line. These detailed results were not obtained by analyses with the 48 markers. Carlsson (2008) demonstrated accurate genetic clustering of chicken populations with 20 markers presenting *F_ST_* values greater than 0.1. In Rosenberg et al. (2001), when 18‒21 markers showing high *NA*, *He* or *F_ST_* values were examined for 30 chickens from each population, clustering accuracy was higher than 98%. Therefore, the present result shows that the two sets of 20 highly polymorphic microsatellite markers (NeHe‐20 and F_ST_‐20), presenting high *Ne*, *He* and *F_ST_* values and low null allele frequencies, are effective for estimating the genetic diversity of farm quail populations and their genetic relationships.

## 5 CONCLUSIONS

Japanese quail are expected to become more widespread in developing countries as a useful source of animal protein. When breeding programmes are formulated to improve egg and/or meat production efficiency of poultry, genetic assessment of farm populations will be important to avoid the loss of genetic diversity or the accumulation of homozygosity that could result in decreased productivity. In this study, using the 50 microsatellite markers, we performed a range of molecular phylogenetic and STRUCTURE analyses that clearly demonstrated the breeding histories and genetic diversities of both the laboratory lines and farm populations of quail from the Aichi prefecture, Japan. The two laboratory quail lines, the AARC‐BB and AARC‐WW, were found to be genetically relatively distant from the farm populations. Based on our data, the Aichi Agricultural Research Center has started to introduce the two laboratory lines into farm populations in Toyahashi city. Furthermore, 20 highly polymorphic microsatellite markers selected from among the 48 markers also accurately estimated the genetic diversity of the quail populations and their genetic relationships, indicating that these microsatellite markers are useful for the genetic assessment of farm quail populations.

## AUTHOR CONTRIBUTION

**Mitsuo Nunome:** Data curation; Formal analysis; Investigation; Methodology; Resources; Validation; Visualization; Writing‐original draft. **Rie Yoshioka:** Data curation; Investigation; Resources. **Takuro Shinkai:** Data curation; Formal analysis; Investigation; Validation; Visualization. **Katsutoshi Kino:** Data curation; Project administration; Resources; Supervision; Writing‐review & editing. **Yoichi Matsuda:** Conceptualization; Project administration; Resources; Supervision; Writing‐original draft; Writing‐review & editing.

### Peer Review

The peer review history for this article is available at https://publons.com/publon/10.1002/vms3.328.

## Supporting information

Table S1
